# Supplementary material for: Enteric Pathogen Testing Importance for Children with Acute Gastroenteritis: a Modified Delphi Study
Source: Microbiol Spectr. 2022 Sep 20;10(5):e01864-22. doi: 10.1128/spectrum.01864-22 (PMC9602993; doi:10.1128/spectrum.01864-22)
Supplement: Supplemental file 1 — Download spectrum.01864-22-s0001.pdf, PDF file, 0.8 MB [file spectrum.01864-22-s0001.pdf]

Enteric Pathogen Testing Importance for Children with Acute Gastroenteritis: A Modified Delphi Study

Supplemental Material

Contents

Supplemental Tables 2
Appendix 1: Round 1 Survey 5
Appendix 2: Round 3 Survey 9
Appendix 3: Round 1 Comments 13
Appendix 4: Round 2 Themes 20
Appendix 5: Round 3 Comments 22

Supplemental Tables

Table S1. Percent agreement among public health practitioners for recommendation of testing of enteropathogens in Rounds 1 and 3 for public health purposes.

	Round 1	*Round 3*
***Aeromonas* spp.**	0%	-
***Campylobacter* spp.**	100%	↑ 100%
C. difficile	40%	0%
***E. coli* O157**	100%	100%
**STEC**	80%	100%
**ETEC**	↑ 60%	0%
***Plesiomonas* spp.**	-	0%
***Salmonella* spp.**	80%	100%
***Shigella* spp.**	80%	100%
***Vibrio* spp.**	↑ 100%	100%
***Yersinia* spp.**	↑ 80%	↑ 100%
***Cryptosporidium* spp.**	100%	↑ 100%
***Cyclospora* spp.**	-	↑ 100%
E. histolytica	40%	0%
***Giardia* spp.**	60%	0%
**Adenovirus 40/41**	↑ 60%	↑ 100%
**Adenovirus non-40/41**	20%	0%
**Astrovirus**	40%	↑ 100%
**Norovirus**	↑ 80%	100%
**Rotavirus**	60%	100%
**Sapovirus**	40%	↑ 100%
**All negative**	20%	0%

Abbreviations: ETEC, enterotoxigenic *E. coli*; IC, immunocompromised; NIC, non-immunocompromised; STEC, Shiga toxin-producing *E. coli*

Bright green shading indicates a high level of agreement, ≥80%. Light green shading indicates a moderate level of agreement, ≥60% to <80%.

↑↓ Arrows indicate whether public health practitioners' level of agreement (e.g. high, moderate) was greater or less than that of all participants in the same round, shown in Table 2.

Table S2. Percent agreement among clinicians for recommendation of testing of enteropathogens in Rounds 1 and 3 for clinical management, by immune status.

	Round 1	*Round 3 – IC*	*Round 3 – NIC*
***Aeromonas* spp.**	8%	-	-
***Campylobacter* spp.**	100%	100%	89%
C. difficile	100%	↓ 78%	↓ 56%
***E. coli* O157**	92%	100%	89%
**STEC**	92%	100%	89%
**ETEC**	42%	56%	44%
***Plesiomonas* spp.**	-	33%	33%
***Salmonella* spp.**	100%	100%	89%
***Shigella* spp.**	92%	100%	89%
***Vibrio* spp.**	↓ 50%	78%	67%
***Yersinia* spp.**	↓ 67%	89%	78%
***Cryptosporidium* spp.**	↓ 67%	89%	67%
***Cyclospora* spp.**	-	67%	22%
E. histolytica	67%	↑ 89%	78%
***Giardia* spp.**	92%	100%	↑ 89%
**Adenovirus 40/41**	↓ 58%	67%	44%
**Adenovirus non-40/41**	17%	44%	0%
**Astrovirus**	33%	33%	22%
**Norovirus**	↓ 58%	67%	56%
**Rotavirus**	↓ 50%	67%	56%
**Sapovirus**	33%	44%	33%
**All negative**	67%	↑ 88%	

Abbreviations: ETEC, enterotoxigenic *E. coli*; IC, immunocompromised; NIC, non-immunocompromised; STEC, Shiga toxin-producing *E. coli*

Bright green shading indicates a high level of agreement, ≥80%. Light green shading indicates a moderate level of agreement, ≥60% to <80%.

↑↓ Arrows indicate whether clinicians' level of agreement (e.g. high, moderate) was greater or less than that of all participants in the same round, shown in Table 2.

Table S3. Differences in percent agreement among all participants for recommendation of testing of enteropathogens in Round 3 for clinical management by immune status.

	Round 3 – IC	*Round 3 – NIC*	*Difference*	*p-value*
Campylobacter spp.	93%	87%	6%	0.710
C. difficile	87%	67%	20%	0.167
E. coli O157	93%	87%	6%	0.710
STEC	100%	93%	7%	0.620
ETEC	47%	40%	7%	0.608
Plesiomonas spp.	29%	29%	0%	1.000
Salmonella spp.	93%	87%	6%	0.710
Shigella spp.	100%	93%	7%	0.620
Vibrio spp.	73%	67%	6%	0.796
Yersinia spp.	87%	73%	6%	0.382
Cryptosporidium spp.	86%	64%	12%	0.103
Cyclospora spp.	71%	36%	35%	0.005
E. histolytica	79%	71%	8%	0.576
Giardia spp.	86%	79%	7%	0.751
Adenovirus 40/41	71%	43%	38%	0.033
Adenovirus non-40/41	50%	0%	50%	<0.001
Astrovirus	29%	14%	15%	0.148
Norovirus	71%	50%	21%	0.119
Rotavirus	79%	57%	22%	0.115
Sapovirus	43%	29%	14%	0.394

Abbreviations: ETEC, enterotoxigenic *E. coli*; IC, immunocompromised; NIC, non-immunocompromised; STEC, Shiga toxin-producing *E. coli*

Exact binomial tests were used to compare IC and NIC proportions.

Appendix 1: Round 1 Survey

For pediatric patients presenting for outpatient care with acute gastroenteritis, please indicate for which pathogens you believe laboratory testing should be recommended to inform clinical management or public health interventions. You may also offer a rationale for your choice, which will be anonymized and compiled with other responses and reported back for discussion during Round 2.

Bacteria

Laboratory testing of ***Aeromonas* spp.** desired for…	Clinical Management	Public Health
	□ Yes	□ Yes
	□ No	□ No
	□ Unsure	□ Unsure
Comments *(optional)*:		
Laboratory testing of ***Campylobacter* spp.** desired for…	Clinical Management	Public Health
	□ Yes	□ Yes
	□ No	□ No
	□ Unsure	□ Unsure
Comments *(optional)*:		
Laboratory testing of ***Clostridium difficile*** desired for…	Clinical Management	Public Health
	□ Yes	□ Yes
	□ No	□ No
	□ Unsure	□ Unsure
Comments *(optional)*:		
Laboratory testing of ***Escherichia coli* O157** desired for…	Clinical Management	Public Health
	□ Yes	□ Yes
	□ No	□ No
	□ Unsure	□ Unsure
Comments *(optional)*:		
Laboratory testing of **Shiga toxin-producing *E. coli* (STEC)** desired for…	Clinical Management	Public Health
	□ Yes	□ Yes
	□ No	□ No
	□ Unsure	□ Unsure
Comments *(optional)*:		
Laboratory testing of **enterotoxigenic *E. coli* (ETEC)** desired for…	Clinical Management	Public Health
	□ Yes	□ Yes
	□ No	□ No
	□ Unsure	□ Unsure
Comments *(optional)*:		
Laboratory testing of ***Salmonella* spp.** desired for…	Clinical Management	Public Health
	□ Yes	□ Yes
	□ No	□ No

	☐ Unsure	☐ Unsure
Comments *(optional)*:		
Laboratory testing of ***Shigella* spp.** desired for…	Clinical Management	Public Health
	☐ Yes	☐ Yes
	☐ No	☐ No
	☐ Unsure	☐ Unsure
Comments *(optional)*:		
Laboratory testing of ***Vibrio* spp.** desired for…	Clinical Management	Public Health
	☐ Yes	☐ Yes
	☐ No	☐ No
	☐ Unsure	☐ Unsure
Comments *(optional)*:		
Laboratory testing of ***Yersinia* spp.** (excluding *Y. pestis)* desired for…	Clinical Management	Public Health
	☐ Yes	☐ Yes
	☐ No	☐ No
	☐ Unsure	☐ Unsure
Comments *(optional)*:		
Are there any bacterial enteropathogens not listed above for which laboratory testing is desired?		

Viruses

Laboratory testing of **adenovirus, types 40/41** desired for…	Clinical Management	Public Health
	☐ Yes	☐ Yes
	☐ No	☐ No
	☐ Unsure	☐ Unsure
Comments *(optional)*:		
Laboratory testing of **adenovirus, other types** desired for…	Clinical Management	Public Health
	☐ Yes	☐ Yes
	☐ No	☐ No
	☐ Unsure	☐ Unsure
Comments *(optional)*:		
Laboratory testing of **astrovirus** desired for…	Clinical Management	Public Health
	☐ Yes	☐ Yes
	☐ No	☐ No
	☐ Unsure	☐ Unsure
Comments *(optional)*:		
Laboratory testing of **norovirus** desired for…	Clinical Management	Public Health
	☐ Yes	☐ Yes
	☐ No	☐ No
	☐ Unsure	☐ Unsure

Comments *(optional)*:		
Laboratory testing of **rotavirus** desired for…	Clinical Management	Public Health
	☐ Yes	☐ Yes
	☐ No	☐ No
	☐ Unsure	☐ Unsure
Comments *(optional)*:		
Laboratory testing of **sapovirus** desired for…	Clinical Management	Public Health
	☐ Yes	☐ Yes
	☐ No	☐ No
	☐ Unsure	☐ Unsure
Comments *(optional)*:		
Are there any viral enteropathogens not listed above for which laboratory testing is desired?		

Parasites

Laboratory testing of ***Cryptosporidium* spp.** desired for…	Clinical Management	Public Health
	☐ Yes	☐ Yes
	☐ No	☐ No
	☐ Unsure	☐ Unsure
Comments *(optional)*:		
Laboratory testing of ***Entamoeba histolytica*** desired for…	Clinical Management	Public Health
	☐ Yes	☐ Yes
	☐ No	☐ No
	☐ Unsure	☐ Unsure
Comments *(optional)*:		
Laboratory testing of ***Giardia* spp.** desired for…	Clinical Management	Public Health
	☐ Yes	☐ Yes
	☐ No	☐ No
	☐ Unsure	☐ Unsure
Comments *(optional)*:		
Are there any parasitic enteropathogens not listed above for which laboratory testing is desired?		

Negative for all pathogens

Is laboratory testing sometimes desired if a child is likely to test negative for all pathogens (e.g. as a rule-out)?	Clinical Management	Public Health
	☐ Yes	☐ Yes
	☐ No	☐ No
	☐ Unsure	☐ Unsure

Comments *(optional)*:	

Appendix 2: Round 3 Survey

For pediatric patients presenting for outpatient care with acute gastroenteritis, please indicate for which pathogens you believe laboratory testing should be recommended to inform clinical management or public health interventions. You may also offer a rationale for your choice, which will be anonymized and compiled with other responses.

Bacteria

Laboratory testing of ***Campylobacter* spp.** desired for…	Clinical Management	Public Health
	□ Yes	□ Yes
	□ No	□ No
	□ Unsure	□ Unsure
Comments *(optional)*:	□ Recommended only for immunocompromised patients	
Laboratory testing of ***Clostridium difficile*** desired for…	Clinical Management	Public Health
	□ Yes	□ Yes
	□ No	□ No
	□ Unsure	□ Unsure
Comments *(optional)*:	□ Recommended only for immunocompromised patients	
Laboratory testing of ***Escherichia coli* O157** desired for…	Clinical Management	Public Health
	□ Yes	□ Yes
	□ No	□ No
	□ Unsure	□ Unsure
Comments *(optional)*:	□ Recommended only for immunocompromised patients	
Laboratory testing of **Shiga toxin-producing *E. coli* (STEC)** desired for…	Clinical Management	Public Health
	□ Yes	□ Yes
	□ No	□ No
	□ Unsure	□ Unsure
Comments *(optional)*:	□ Recommended only for immunocompromised patients	
Laboratory testing of **enterotoxigenic *E. coli* (ETEC)** desired for…	Clinical Management	Public Health
	□ Yes	□ Yes
	□ No	□ No
	□ Unsure	□ Unsure
Comments *(optional)*:	□ Recommended only for immunocompromised patients	
Laboratory testing of ***Salmonella* spp.** desired for…	Clinical Management	Public Health
	□ Yes	□ Yes
	□ No	□ No
	□ Unsure	□ Unsure

Comments *(optional)*:	◻ Recommended only for immunocompromised patients	
Laboratory testing of ***Shigella* spp.** desired for…	Clinical Management	Public Health
	◻ Yes	◻ Yes
	◻ No	◻ No
	◻ Unsure	◻ Unsure
Comments *(optional)*:	◻ Recommended only for immunocompromised patients	
Laboratory testing of ***Vibrio* spp.** desired for…	Clinical Management	Public Health
	◻ Yes	◻ Yes
	◻ No	◻ No
	◻ Unsure	◻ Unsure
Comments *(optional)*:	◻ Recommended only for immunocompromised patients	
Laboratory testing of ***Yersinia* spp.** (excluding *Y. pestis)* desired for…	Clinical Management	Public Health
	◻ Yes	◻ Yes
	◻ No	◻ No
	◻ Unsure	◻ Unsure
Comments *(optional)*:	◻ Recommended only for immunocompromised patients	
Laboratory testing of ***Plesiomonas*** desired for…	Clinical Management	Public Health
	◻ Yes	◻ Yes
	◻ No	◻ No
	◻ Unsure	◻ Unsure
Comments *(optional)*:	◻ Recommended only for immunocompromised patients	

Viruses

Laboratory testing of **adenovirus, types 40/41** desired for…	Clinical Management	Public Health
	◻ Yes	◻ Yes
	◻ No	◻ No
	◻ Unsure	◻ Unsure
Comments *(optional)*:	◻ Recommended only for immunocompromised patients	
Laboratory testing of **adenovirus, other types** desired for…	Clinical Management	Public Health
	◻ Yes	◻ Yes
	◻ No	◻ No
	◻ Unsure	◻ Unsure
Comments *(optional)*:	◻ Recommended only for immunocompromised patients	
Laboratory testing of **astrovirus** desired for…	Clinical Management	Public Health
	◻ Yes	◻ Yes

	☐ No	☐ No
	☐ Unsure	☐ Unsure
Comments *(optional)*:	☐ Recommended only for immunocompromised patients	
Laboratory testing of **norovirus** desired for…	Clinical Management	Public Health
	☐ Yes	☐ Yes
	☐ No	☐ No
	☐ Unsure	☐ Unsure
Comments *(optional)*:	☐ Recommended only for immunocompromised patients	
Laboratory testing of **rotavirus** desired for…	Clinical Management	Public Health
	☐ Yes	☐ Yes
	☐ No	☐ No
	☐ Unsure	☐ Unsure
Comments *(optional)*:	☐ Recommended only for immunocompromised patients	
Laboratory testing of **sapovirus** desired for…	Clinical Management	Public Health
	☐ Yes	☐ Yes
	☐ No	☐ No
	☐ Unsure	☐ Unsure
Comments *(optional)*:	☐ Recommended only for immunocompromised patients	

Parasites

Laboratory testing of ***Cryptosporidium* spp.** desired for…	Clinical Management	Public Health
	☐ Yes	☐ Yes
	☐ No	☐ No
	☐ Unsure	☐ Unsure
Comments *(optional)*:	☐ Recommended only for immunocompromised patients	
Laboratory testing of ***Entamoeba histolytica*** desired for…	Clinical Management	Public Health
	☐ Yes	☐ Yes
	☐ No	☐ No
	☐ Unsure	☐ Unsure
Comments *(optional)*:	☐ Recommended only for immunocompromised patients	
Laboratory testing of ***Giardia* spp.** desired for…	Clinical Management	Public Health
	☐ Yes	☐ Yes
	☐ No	☐ No
	☐ Unsure	☐ Unsure
Comments *(optional)*:	☐ Recommended only for immunocompromised patients	

Laboratory testing of ***Cyclospora*** desired for…	Clinical Management	Public Health
	☐ Yes	☐ Yes
	☐ No	☐ No
	☐ Unsure	☐ Unsure
Comments *(optional)*:	☐ Recommended only for immunocompromised patients	

Negative for all pathogens

Is laboratory testing sometimes desired if a child is likely to test negative for all pathogens (e.g. as a rule-out)?	Clinical management	Public health
	☐ Yes	☐ Yes
	☐ No	☐ No
	☐ Unsure	☐ Unsure
Comments *(optional)*:		

Appendix 3: Round 1 Comments

Additional Pathogens

Respondents indicated these pathogens should be considered for testing. For some pathogens, testing was recommended only in the presence of specific risk factors.

Pathogen Type	Pathogen
Bacteria	*Cronobacter sakazkii*
	Edwardsiella
	Plesiomonas (3)
	EAEC
	Listeria monocytogenes
Viruses	Human bocaviruses
Parasites	*Ascaris*
	Enterobius vermicularis (pinworm)
	Cyclospora (5)
	Isospora
	Strongyloides stercoralis
	D. fragilis
	B. hominis

Comments

Bacteria

Aeromonas spp.

Response	Clinical Rationale	Public Health Rationale
Yes	• There is a sentiment that this is a pathogen. Excluding it often enables us to move forward with other diagnoses, such as inflammatory bowel disease.	
No	• Maybe for immunocompromised only	• Does not appear to have major public health impact.<br>• Essentially no single strain outbreaks reported. Small number of possible outbreaks.<br>• Invasive infections (sterile site cultures) will be captured without testing stool<br>• Outbreaks are rare, but could occur. If we identify a cluster, that would help strengthen the case for *Aeromonas* is a pathogen. There is always a potential it

		will lead to a recall/improved bactericidal processes.
Unsure	• Some reports indicate *Aeromonas* can cause symptoms while others are less convincing.<br>• No clear evidence of pathogenicity in terms of acute gastroenteritis.	

Campylobacter spp.

Response	Clinical Rationale	Public Health Rationale
Yes	• Sometimes helpful to inform treatment<br>• Treatment, early on, can be beneficial.<br>• Even though majority of time antibiotics is not indicated as many patients only have mild gastroenteritis illness, testing for Campylobacter is also indicated are other non-gastroenteritis clinical presentations e.g., Guillain-Barré syndrome.<br>• I would treat.	• Reportable disease<br>• Only culture confirmed (not PCR only) and sometimes useful in outbreak scenarios<br>• Follow-up with questionnaire for detection of clusters
No		• Rarely causes outbreaks.<br>• Most cases are sporadic. There could be surveillance value, but not in individual cases.
Unsure		

C. difficile

Response	Clinical Rationale	Public Health Rationale
Yes	• If an inpatient or facility resident, clinical context details required<br>• Not under 2 years of age<br>• Treatment can be life saving.<br>• But the relevance of testing is age and clinical picture dependent<br>• Diagnostic stewardship is imperative for everything but particularly for C diff<br>• Would want to know to determine contact management<br>• Age-dependent.<br>• IPC precaution and isolation	• Not under 2 years of age<br>• Can cause outbreaks.<br>• But the relevance of testing is age and clinical picture dependent<br>• To help us understand a less-understood bacterium, including issues around asymptomatic cultures, and even whether there is any benefit from their presence at certain ages
No	• Not informative for children <2 years, perhaps <3 years	• Surveillance only in hospital.
Unsure		

E. coli O157

Response	Clinical Rationale	Public Health Rationale
Yes	• Severe disease.<br>• Determine whether to give antibiotics<br>• Self-evident	• Reportable disease<br>• Highly transmissible.<br>• Self-evident<br>• Cases follow-up and also for microbiological clearance especially in certain profession and also children attending day/child care facilities or schools
No		
Unsure		

Shiga toxin-producing *E. coli* (STEC)

Response	Clinical Rationale	Public Health Rationale
Yes	• Non-O157 can also cause HUS.<br>• Yes, especially if *stx* genotype is provided.	• Reportable disease<br>• Non-O157 can cause outbreaks.<br>• Almost none for an *stx1+/stx2-* STEC. More important for high risk STEC (*stx2+* or *stx1+/stx2+*)<br>• Cases follow-up and also for microbiological clearance especially in certain profession and also children attending day/child care facilities or schools
No		• Clinical significance not established for PCR only
Unsure		

Enterotoxigenic *E. coli* (ETEC)

Response	Clinical Rationale	Public Health Rationale
Yes	• Can be associated with severe disease particularly in younger children and some evidence of antibiotic treatment benefit in travellers.	• Reportable disease
No	• In right context in North America, i.e., returned traveller	• Public health implications in N. America are unclear.<br>• None
Unsure	• Can be important in returning traveler but otherwise not usually helpful.	

***Salmonella* spp.**

Response	Clinical Rationale	Public Health Rationale
Yes	• Antibx sometimes indicated<br>• Can explain symptoms and also lead to disseminated disease.	• Highly contagious.<br>• Reportable disease<br>• Cases follow-up and also for microbiological clearance especially in certain profession and also children attending day/child care facilities or schools
No		• Does not change PH management. Only culture confirmed not PCR only. Often the patients are already 48 hrs asymptomatic when reported so exclusions are not necessary.
Unsure		

***Shigella* spp.**

Response	Clinical Rationale	Public Health Rationale
Yes	• Can lead to severe disease.<br>• Determine appropriate antibiotics	• Reportable disease<br>• Cases follow-up and also for microbiological clearance especially in certain profession and also children attending day/child care facilities or schools
No		• Does not change PH management. Only culture confirmed not PCR only. Often the patients are already 48 hrs asymptomatic when reported so exclusions are not necessary.
Unsure	• Does not change management	

***Vibrio* spp.**

Response	Clinical Rationale	Public Health Rationale
Yes	• Helpful for supportive management<br>• Can lead to severe symptoms.<br>• But only when there is significant risk exposure from clinical history	• Reportable, all travel related<br>• Reportable disease<br>• Notifiable for foodborne outbreak purpose
No	• Cholera unheard of in the US. Not a priority for routine care in the non-foreign traveler	• Invasive Vibrio infections have public health performance
Unsure	• Probably not in developed countries, maybe only in the setting of symptoms and epidemic	

Yersinia spp.

Response	Clinical Rationale	Public Health Rationale
Yes	• But only for certain clinical presentation	• Reportable disease
No	• Rarely clinically important.	• Only helpful for outbreak scenarios (MSM in the past)<br>• No associated outbreaks.
Unsure		

Viruses

Adenovirus 40/41

Response	Clinical Rationale	Public Health Rationale
Yes	• Helps clarify treatment.<br>• To rule out the other pathogens<br>• Clinical management is more relevant for certain patients, e.g., organ transplant and immunocompromised patients<br>• If there is a cluster of unexplained illnesses with hospitalization<br>• A positive result offers clarity.	• We did have an outbreak of D41 associated with increased morbidity and mortality
No		• Surveillance value only.
Unsure		

Adenovirus (other)

Response	Clinical Rationale	Public Health Rationale
Yes	• Clinical management is more relevant for certain patients, e.g., organ transplant and immunocompromised patients	
No	• May not represent enteric infection.	• Surveillance value only.
Unsure	• Yes if hospitalized<br>• Awaiting data.	

Astrovirus

Response	Clinical Rationale	Public Health Rationale
Yes	• A positive result offers clarity.<br>• Yes in certain population, e.g., immunocompromised patients	
No	• Uncommon.	• Surveillance value only.<br>• For surveillance only
Unsure		

Norovirus

Response	Clinical Rationale	Public Health Rationale
Yes	• Helps clarify treatment.<br>• A positive result offers clarity.	• Only helpful for outbreak precautions<br>• Outbreaks and vaccine program candidate.
No		• Surveillance value only.<br>• For surveillance only
Unsure		

Rotavirus

Response	Clinical Rationale	Public Health Rationale
Yes	• Helps clarify treatment.	• Outbreaks and vaccine program available.<br>• Vaccine preventable disease
No	• A positive result offers clarity.	• Surveillance value only.<br>• For surveillance only
Unsure		

Sapovirus

Response	Clinical Rationale	Public Health Rationale
Yes	• Helps clarify treatment.<br>• A positive result offers clarity.	
No		• Surveillance value only.
Unsure		

Parasites

Cryptosporidium spp.

Response	Clinical Rationale	Public Health Rationale
Yes	• Clinically relevant requiring specific treatment.<br>• Treatable. A positive result might not reflect the cause of the diarrhea as there is asymptomatic carriage in the community.	• Associated with outbreaks.<br>• Reportable illness in Oregon<br>• Outbreaks happen. Helpful for surveillance.
No		
Unsure		

Entamoeba histolytica

Response	Clinical Rationale	Public Health Rationale

Yes	• Clinically relevant requiring specific treatment.	• Very rare outbreaks in NA
No		• All travel related<br>• Not associated with outbreaks.<br>• Not reportable
Unsure	• Treatable but incredibly rare in NA (except parts of Mexico).	

Giardia spp.

Response	Clinical Rationale	Public Health Rationale
Yes	• Clinically relevant requiring specific treatment.<br>• Treatable. A positive result might not reflect the cause of the diarrhea as there is asymptomatic carriage in the community.	• Reportable illness in Oregon<br>• Outbreaks are rare. Helpful for surveillance.
No		• Not really, does not change PH management. Often the patients are already 48 hrs asymptomatic when reported so exclusions are not necessary.<br>• Not associated with outbreaks.
Unsure		

All Negative

Response	Clinical Rationale	Public Health Rationale
Yes	• Rarely<br>• If all negative can make you wonder if etiology is non-enteric infection.<br>• High risk patients for severe disease (e.g. BMT) and also for patients being admitted to hospital who have other like explanations (e.g. chemotherapy) but there are infection control implications.<br>• May be value to rule out an infectious cause before justifying an expensive work up for unusual non-infectious process that may include invasive and/or other harmful procedures<br>• This will enable return to, for example, day care after post-symptomatic carriage is excluded. So, it is sort of clinical management.	
No		• A negative culture is no substitute for good environmental hygiene.
Unsure		

Appendix 4: Round 2 Themes

Aeromonas and Non-40/41 Adenovirus

- Testing for these pathogens would not change clinical management and is therefore unnecessary, with the exception of adenovirus in immunocompromised patients
- Results could be interesting and if provided should be coupled with a directive report

ETEC

- ETEC is now reportable to public health in some U.S. states
- The burden and epidemiology of ETEC in the U.S. and Canada is largely unknown, but recent testing suggests it is more common than perhaps previously thought
- Testing and reporting cases could facilitate outbreak investigations
- Testing would not change clinical management but could inform differential diagnosis
- Testing may only be necessary in select cases

Astrovirus and Sapovirus

- Testing would not change clinical management but could inform differential diagnosis
- Given its potential for outbreaks, sapovirus should be considered for testing, particularly among immunocompromised patients

E. coli O157 and Other STEC

- Testing for these is a high priority, because of treatment guidelines (antibiotics are contraindicated)
- The stx genotype would add value to STEC testing results by guiding the aggressiveness of clinical management and public health interventions
- However, testing should still be conducted even if the stx genotype is unknown, because of the risk associated with antibiotic administration

Salmonella

- Opinions are mixed as to whether the serotype is necessary for the relevance of Salmonella testing

Testing Only Under Specific Circumstances

- For pathogens that only require testing under specific circumstances (e.g. immunocompromised patients), the recommendation to test should only be made in that circumstance
- However, if bloody diarrhea is present, testing should be recommended regardless of specific circumstances

Appendix 5: Round 3 Comments

Bacteria

Campylobacter spp.

Response	Clinical Rationale	Public Health Rationale
Yes	• Helpful to identify cause of bloody diarrhea, and occasionally requires treatment.<br>• Antibiotics generally not required, but useful in shortening duration of illness if given early in course of illness.<br>• Even though most of the time specific treatment is not required, it is helpful to have a clinical diagnosis made and point source might be identified and better food handling practice can be communicated.	• Outbreaks not commonly identified, but do occur.<br>• Clustering of cases and point source might be identified for outbreak investigation, control, and prevention.
Only IC		N/A
No		• Outbreaks are rare.
Unsure		

C. difficile

Response	Clinical Rationale	Public Health Rationale
Yes	• Testing is very helpful in at-risk children (sick, hospitalized kids, a lot of antibiotics) but should not be routinely performed on outpatients.<br>• For children this should only be reported if requested by the clinician (i.e. there are identified risk factors) given the high rate of colonization.<br>• For adults [only].<br>• Gate based on age (no testing <2 y.o.).<br>• Age restriction required.	• Important to monitor trends and spread of true disease.
Only IC		N/A
No		
Unsure	• Highly age and clinical context dependent.<br>• Very age dependent because of asymptomatic carriage in young infants; not a simple yes, and appropriate history including antibiotic exposure needs to be obtained.	• For hospital infection control measures, not necessarily "public health."<br>• Outbreaks can occur but mainly in acute care settings, but having the overall data is important for both infection prevention and control and antimicrobial stewardship programs.

E. coli O157

Response	Clinical Rationale	Public Health Rationale
Yes	• Detection will influence clinical management.<br>• Highest importance.<br>• Possible clinical complications with severe morbidity and associated mortality. Moreover, having a clinical diagnosis made might identify point source resulting in food safety and better food handling practice. Advancement in diagnostic testing should be employed to identify STEC organisms, especially *stx2* beyond detection of *E. coli* O157. The serotyping is still important for source tracking.	• Outbreaks can be devastating.<br>• Clustering of cases and point source might be identified for outbreak investigation, control and prevention. Advancement in diagnostic testing should be employed to identify STEC organisms, especially *stx2* beyond detection of *E. coli* O157. The serotyping is still important for source tracking.
Only IC		N/A
No		
Unsure	• Specific testing for O157 not necessary if stx subtype available by culture or PCR.	

Shiga toxin-producing *E. coli* (STEC)

Response	Clinical Rationale	Public Health Rationale
Yes	• Caveats - if clinical context is right, absolutely. If not, no. If resulted as part of a panel, be skeptical. Ideally, high risk vs. low risk should be provided by genotyping the STEC (*stx2* = high risk).<br>• Detection will influence clinical management.<br>• Highest importance.<br>• Having the ability to distinguish between *stx1* and *stx2* is important for both clinical management of cases and public health follow up because of the different risk associated with adverse complications and outcomes.	• Outbreaks can be devastating.<br>• Having the ability to distinguish between *stx1* and *stx2* is important for both clinical management of cases and public health follow up because of the different risk associated with adverse complications and outcomes.
Only IC		N/A
No		
Unsure		

Enterotoxigenic *E. coli* (ETEC)

Response	Clinical Rationale	Public Health Rationale

Yes		
Only IC		N/A
No	• No clinical relevance outside of travelers.<br>• Usually mild illness, non-outbreak associated.	• No clinical relevance outside of travelers.<br>• Usually mild illness, non-outbreak associated.
Unsure	• In returned travelers, helpful. Others, much less so.	

Plesiomonas spp.

Response	Clinical Rationale	Public Health Rationale
Yes		
Only IC		N/A
No	• Unknown clinical relevance.	• Does not tend to cause outbreaks.
Unsure		

Salmonella spp.

Response	Clinical Rationale	Public Health Rationale
Yes	• Can lead to severe disease and detection can require treatment.<br>• Often, the children do not require treatment but we cannot be sure until we know their results and have called them back to check on them.<br>• More severe, invasive infections can occur in low proportion of patients.<br>• Depending on age and Typhoid versus non-Typhoid strains, the risk of invasive disease is different, and antibiotics might be needed.	• Important to prevent outbreaks.<br>• Clustering of cases and point source might be identified for outbreak investigation, control, and prevention.
Only IC		N/A
No		
Unsure		

Shigella spp.

Response	Clinical Rationale	Public Health Rationale
Yes	• Can lead to severe disease and detection can require treatment.<br>• Clinicians should be aware that this is an increasing problem among MSM and homeless.<br>• Antibiotic management decreases spread and improves symptoms.	• Important to prevent outbreaks.<br>• MDR shigella is increasingly common and needing to be monitored.<br>• Clustering of cases and point source might be identified for outbreak investigation, control and prevention.

Only IC		N/A
No		
Unsure		

Vibrio spp.

Response	Clinical Rationale	Public Health Rationale
Yes	• This is probably not needed except in certain endemic areas or returned travelers. Non-cholera vibrios might be considered in immunocompromised.<br>• Only for patients with exposure risk.	• Clustering of cases and point source might be identified for outbreak investigation, control and prevention
Only IC		N/A
No	• Rare in AB context and not usually requiring treatment.<br>• Beware of false positives with multipathogen assay tests.	• Does not tend to cause outbreaks.
Unsure		

Yersinia spp.

Response	Clinical Rationale	Public Health Rationale
Yes	• Increasingly detecting cases that would've been missed before: some present as pseudo-appendicitis, and some systemically infected with culture+ breakthrough sores long after diarrhea stopped.<br>• A cause of "pseudo-appendicitis", so is helpful to know if this might be causing those symptoms.<br>• Only for specific clinical presentation.<br>• Age restriction.	• Increasingly detected, now that many clinical labs are using multipathogen assays. Epidemiological understanding of who gets Yersinia is changing (and what cases are related, through WGS) and relatively large outbreaks (n=100) were detected in the US in 2019.<br>• Only for specific settings.
Only IC		N/A
No	• Unknown or limited clinical relevance.	• Does not tend to cause outbreaks.
Unsure		

Viruses

Adenovirus 40/41

Response	Clinical Rationale	Public Health Rationale
Yes	• Common cause of gastroenteritis and detection can inform management.	• Gastroenteritis outbreak infection and control.

		• Determine cause of outbreaks in non-health care settings for direct intervention or policy changes to prevent additional or future infections.
Only IC	• Adenovirus 40/41 can cause sever gastroenteritis, especially in young children and elders, especially in immunocompromised. managements include reduction of immunosuppressive drugs, intravenous immunoglobulin, and intravenous antiviral therapy with cidofovir.<br>• Only immunocompromised.<br>• Can be useful for differential diagnosis and immunocompromised patients.	N/A
No	• Might avert further workup.<br>• Diagnostics always guide clinical management - reassurance, certainty around diagnosis, etc., but may not "change" management.	• No significant PH impact at present.
Unsure		

Adenovirus (other)

Response	Clinical Rationale	Public Health Rationale
Yes		• Determine cause of outbreaks in non-health care settings for direct intervention or policy changes to prevent additional or future infections.
Only IC	• Adenoviruses can cause severe disease, including pneumonitis, hemorrhagic cystitis, hepatitis, and disseminated infection associated with high mortality in immunocompromised patients. Managements see above.<br>• Only immunocompromised.<br>• Important for immunocompromised patients and certain serotypes associated with severe respiratory disease.	N/A
No	• Uncommon etiology and likely represents colonization and nasal shedding.<br>• Diagnostics always guide clinical management - reassurance, certainty around diagnosis, etc., but may not "change" management.	• No significant PH impact at present.<br>• Only when there is clustering of severe respiratory illness.
Unsure	• Viral diagnoses rarely change the course of treatment, but can help us avoid	

	unnecessary tests and provide reassurance to the family (and possible avoidance of repeat visits).	

Astrovirus

Response	Clinical Rationale	Public Health Rationale
Yes		• Gastroenteritis outbreak and infection control.<br>• Determine cause of outbreaks in non-health care settings for direct intervention or policy changes to prevent additional or future infections.
Only IC	• Only immunocompromised.<br>• Mild illness and low prevalence but can be useful for differential diagnosis and immunocompromised patients.	N/A
No	• Uncommon etiology and unlikely to influence management.<br>• Diagnostics always guide clinical management - reassurance, certainty around diagnosis, etc., but may not "change" management.	• No significant PH impact at present.
Unsure	• Viral diagnoses rarely changes the course of treatment, but can help us avoid unnecessary tests and provide reassurance to the family (and possible avoidance of repeat visits).	

Norovirus

Response	Clinical Rationale	Public Health Rationale
Yes	• Norovirus can explain a lot of things, so is valuable.<br>• Viral diagnoses rarely change the course of treatment, but can help us avoid unnecessary tests and provide reassurance to the family (and possible avoidance of repeat visits).	• In community surveillance mode only, or for obvious clusters.<br>• Knowledge can inform vaccine policy.<br>• Gastroenteritis outbreak and infection control.<br>• Determine cause of outbreaks in non-health care settings for direct intervention or policy changes to prevent additional or future infections.<br>• Important for outbreaks, vaccine development and monitoring.
Only IC	• Norovirus illness can also become debilitating and life-threatening in immunocompromised patients. Clinical management is challenge because there	N/A

	is no effective antiviral drug available. Reduction of immunosuppressive drugs and intravenous immunoglobulin could be used for the management.<br>• Only immunocompromised.<br>• Most common viral gastroenteritis pathogen with upcoming vaccine development, can be useful for differential diagnosis, and can be important for immunocompromised patient.	
No		
Unsure		

Rotavirus

Response	Clinical Rationale	Public Health Rationale
Yes	• If reassurance is part of clinical care, yes.<br>• Common cause of severe gastroenteritis and detection can inform management.<br>• Viral diagnoses rarely change the course of treatment, but can help us avoid unnecessary tests and provide reassurance to the family (and possible avoidance of repeat visits).	• Knowledge can inform vaccine policy.<br>• Gastroenteritis outbreak and infection control.<br>• Determine cause of outbreaks in non-health care settings for direct intervention or policy changes to prevent additional or future infections.<br>• Vaccine effectiveness and circulating strain monitoring and some importance in outbreaks in institution.
Only IC	• Rotavirus cause severe gastroenteritis in infants, young children, elders, and immunocompromised patients. No effective antiviral drug available. Treatment involves supportive care including rehydration therapy and restoration of electrolyte balance.<br>• Only immunocompromised.<br>• Vaccine preventable illness and still can cause severe gastroenteritis and can be useful for differential diagnosis. Diagnosis of case can identify emerging new strains.	N/A
No	• Diagnostics always guide clinical management - reassurance, certainty around diagnosis, etc., but may not "change" management.	• In community surveillance only.
Unsure		

Sapovirus

Response	Clinical Rationale	Public Health Rationale
Yes	• Common cause of vomiting and detection can inform testing and management.<br>• Viral diagnoses rarely change the course of treatment, but can help us avoid unnecessary tests and provide reassurance to the family (and possible avoidance of repeat visits).	• Importance in outbreaks with varying annual incidence.<br>• Gastroenteritis outbreak and infection control.<br>• Determine cause of outbreaks in non-health care settings for direct intervention or policy changes to prevent additional or future infections.
Only IC	• Only immunocompromised.<br>• Can be useful for differential diagnosis, especially immunocompromised patient.	N/A
No	• Diagnostics always guide clinical management - reassurance, certainty around diagnosis, etc., but may not "change" management.	• No significant PH impact at present.
Unsure		

Parasites

Cryptosporidium spp.

Response	Clinical Rationale	Public Health Rationale
Yes	• Be somewhat wary of finding a positive result - might be carrier.<br>• Detection will inform management.<br>• There is a specific treatment.<br>• Only with prolonged symptoms (e.g., >7 days).	• Outbreaks do occur and certain high risk areas exist (First Nations).<br>• Outbreaks fairly common, restrictions for swimming generally employed.<br>• Clustering of cases associated with outbreaks.
Only IC	• Diagnosis of cases might uncover common source outbreak and important in immunocompromised hosts.	N/A
No		
Unsure		

Cyclospora spp.

Response	Clinical Rationale	Public Health Rationale
Yes	• There is a specific treatment.<br>• Only with prolonged symptoms (e.g., >7 days).	• Foodborne outbreaks being much more commonly identified.<br>• Clustering of cases associated with outbreaks.
Only IC	• In general, this is a very rarely identified pathogen in North America and outside	N/A

	of immunocompromised hosts tends to self-resolve. Would be helpful to have as part of a panel but would likely only be needed in certain circumstances (e.g., traveler).<br>• Diagnosis of cases might uncover common source outbreak and important in immunocompromised hosts.	
No	• Unclear clinical implications.	• Outbreaks uncommon.
Unsure		

Entamoeba histolytica

Response	Clinical Rationale	Public Health Rationale
Yes	• Highly selected situations.<br>• Detection will inform management.<br>• Only with prolonged symptoms (e.g., >7 days). Consider only if travel.<br>• Can be associated with invasive disease require treatment - the difficulty is differentiating between *E. histolytica* vs. *E. dispar*.	• Clustering of cases associated with outbreaks.
Only IC		N/A
No		• Outbreaks uncommon.<br>• Not in Canada.
Unsure		

Giardia spp.

Response	Clinical Rationale	Public Health Rationale
Yes	• Need treatment in symptomatically infected cases.<br>• Detection will inform management.<br>• Only with prolonged symptoms (e.g., >7 days).	• Clustering of cases associated with outbreaks.
Only IC		N/A
No		• Outbreaks uncommon.
Unsure		

All Negative

Response	Clinical Rationale	Public Health Rationale
Yes	• I wish. However, pan-pathogen testing elicits results that sometimes engender	

	more anxiety than clarity, and encourage unnecessary treatment.<br>• Will inform clinician that etiology is likely non-infectious.<br>• Yes, definitely for children being admitted to hospital with diarrhea and other conditions that may be causal (e.g. GI patients) or those with compromised immune systems (e.g. HIV, oncology).<br>• Rule out is important is certain clinical situation, especially immunocompromised or chronic symptoms.<br>• Diagnostics always guide clinical management - reassurance, certainty around diagnosis, etc., but may not "change" management.	
No		
Unsure		
